# Roux-en-Y gastric bypass and laparoscopic sleeve gastrectomy effects on obesity comorbidities: A systematic review and meta-analysis

**DOI:** 10.3389/fsurg.2022.953804

**Published:** 2022-12-02

**Authors:** Salah Alghamdi, Hyder Mirghani, Khalid Alhazmi, Amirah M. Alatawi, Haneen Brnawi, Tariq Alrasheed, Waleed Badoghaish

**Affiliations:** ^1^ Department of Surgery, Faculty of Medicine, University of Tabuk, Tabuk, Saudi Arabia; ^2^ Department of Internal Medicine, Faculty of Medicine, University of Tabuk, Tabuk, Saudi Arabia; ^3^ Department of Pathology, Faculty of Medicine, University of Tabuk, Tabuk, Saudi Arabia; ^4^ Department of Family and Community Medicine, Faculty of Medicine, University of Tabuk, Tabuk, Saudi Arabia

**Keywords:** Roux-en Y gastric bypass, laparoscopic sleeve gastrectomy, obesity-related comorbidities, obesity, comorbidities

## Abstract

Roux-en-Y gastric bypass (LRYGB) and laparoscopic sleeve gastrectomy (LSG) are the most commonly used bariatric procedures. There is an increasing awareness about a comorbidity-based indication for bariatric surgery regardless of weight (metabolic surgery). The best operation to mitigate obesity-associated comorbidities is a matter of controversy. This review is aimed at comparing LRYGB and LSG for the treatment of diabetes, hypertension, dyslipidemias, obstructive sleep apnea (OSA), and gastroesophageal reflux (GERD). We searched PubMed, MEDLINE, SCOPUS, Web of Science, and Cochrane library for articles comparing these two commonly used bariatric approaches. We identified 2,457 studies, 1,468 of which stood after the removal of duplications; from them, 81 full texts were screened and only 16 studies were included in the final meta-analysis. LRYGB was equal weight to LSG for diabetes (*P*-value = 0.10, odd ratio, 1.24, 95% CI, 0.96–1.61, *I*^2^ for heterogeneity = 30%, *P*-value for heterogeneity, 0.14), and OSA (*P*-value = 0.38, odd ratio, 0.79, 95% CI, 0.47–1.33, *I*^2^ for heterogeneity = 0.0%, *P*-value for heterogeneity, 0.98). However, LRYGB was superior to LSG regarding hypertension (*P*-value = 0.009, odd ratio, 1.55, 95% CI, 1.20–2.0, *I*^2^ for heterogeneity = 0.0%, *P*-value for heterogeneity, 0.59), dyslipidemia (odd ratio, 2.18, 95% CI, 1.15–4.16, *P*-value for overall effect, 0.02), and GERD (*P*-value = 0.003, odd ratio, 3.16, 95% CI, 1.48–6.76). LRYGB was superior to LSG for gastroesophageal reflux, hypertension, and dyslipidemia remission. While the two procedures were equal regarding diabetes and obstructive sleep, further reviews comparing LSG, and one anastomosis gastric bypass are recommended.

## Introduction

Obesity is one of the most challenging pandemics worldwide due to its various complications, which include diabetes mellitus, high blood pressure, respiratory disease, dyslipidemia, and cancer. The prevalence of obesity is rising worldwide (1). An intriguing study shows that despite attempting to lose weight in nearly 100% of participants, around two-thirds achieved a weight loss of ≥5%, but only 5% maintained the weight loss for 1 year (2). In addition, only half of the patients are motivated to lose weight (3). Diabetes mellitus is rapidly increasing, the prevalence worldwide is 9.2% and most patients are not reaching the recommended target for fasting plasma glucose, lipid profile, blood pressure, and lifestyles (4, 5). Furthermore, hypoglycemic medications are not without fatal complications including hypoglycemia and risk of falls, especially among the elderly and frail (6). Despite the emergence of novel antidiabetic medications with cardio-renal protection, their use is limited due to the cost and side effects such as toe amputation, osteoporosis, and infections. Furthermore, most patients with diabetes are not controlled (7, 8). There is a recent shift in the indication of bariatric surgery from a certain body mass index (BMI) to comorbidity-based approach where interdisciplinary care by surgeons, endocrinologists or internists, a psychologist, and a dietician is needed before surgery (9).

Although metabolic surgery is a rapidly growing effective measure for obesity treatment, the uptake is small due to the perceived invasive nature and fear of complications. A meta-analysis concluded the effectiveness of endoscopic sleeve gastroplasty (ESG) with a lower rate of side effects (10).

Roux-en-Y gastric bypass and sleeve gastrectomy are the two common bariatric procedures for obesity and comorbidities. Since its introduction by Mason in 1966, Roux-en-Y gastric bypass accounted for 60%–70% of bariatric surgeries in the US. It is both a restrictive and malabsorptive procedure. The procedure is associated with diabetes remission, lower use of antidiabetic medications, and lower body weight and triglyceride compared with the usual care for diabetes (11). Sleeve gastrectomy is a restrictive bariatric surgery, and due to its lower risk, it is increasingly used. Similar outcomes to bypass were observed. The laparoscopic approach through 1-day surgery is now the standard of care due to the lower morbidity and mortality (11).

The remission of obesity comorbidities such as hypertension, diabetes mellitus, dyslipidemias, obstructive sleep apnea (OSA), and gastroesophageal reflux were documented (12, 13). However, comparisons between various types of metabolic surgery on remission are lacking, therefore, this meta-analysis aimed to compare the effects of Roux-en-Y gastric bypass (LRYGB) and sleeve gastrectomy (LSG) on obesity comorbidities.

## Methods

### Eligibility criteria according to PICOS

#### Inclusion criteria

Studies were included if they were randomized controlled trials carried out among adults >18 years and with BMI >27.5 kg/m^2^ with a minimum of 12 months follow-up. The patients were those who underwent LSG or LRYGB without revision or conversion. The search engine was set to English.

#### Exclusion criteria

Retrospective and prospective cohorts, case-control studies, case reports, and case series were not included. Abstracts, opinions, letters, editorials, and expert opinions were also excluded. Studies with revisions or conversions were not included.

### Outcome measures

The outcome measures were diabetes, hypertension, dyslipidemia, gastroesophageal reflux, and OSA remission. Diabetes remission was defined as HbA1c < 6 without diabetes medication for at least 1 year. Hypertension and dyslipidemia remission is normal blood pressure and lipids without drugs. GERD was defined in the presence of either reflux esophagitis (Los Angeles B, C, or D) or increased total acid exposure (>6%), as recommended by Lyon consensus regardless of typical GERD symptoms.

In the present study, we did not concentrate on specific body mass indexes. The BMI of the patients included in the analyzed studies varied widely. In addition, one study included LSG and Banded LRYGB and another one used LSG and OAGB.

### The literature search

**Figure 1:**
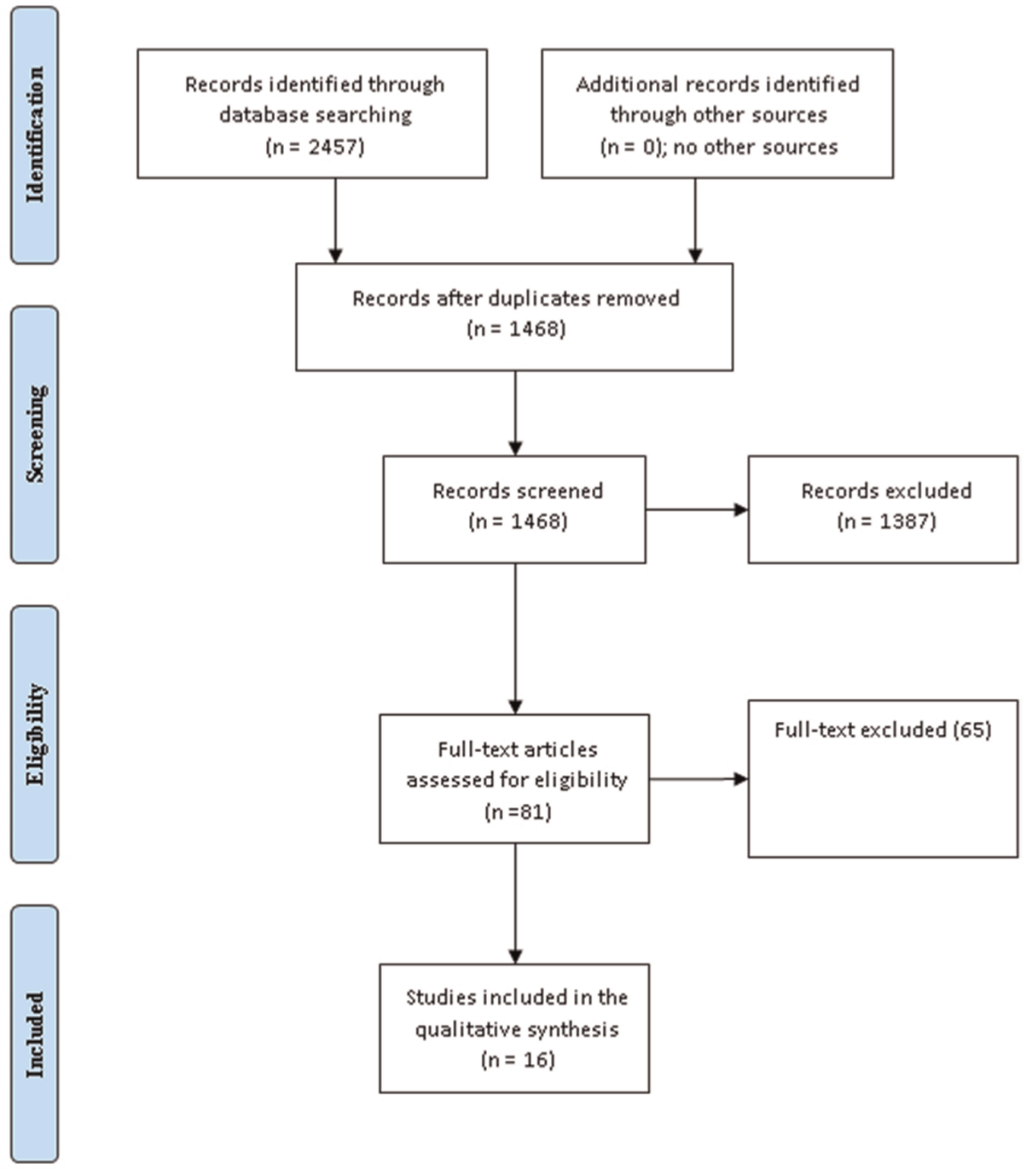
The PRISMA chart (30) for studies comparing Roux-en-Y gastric bypass (LRYGB) and sleeve gastrectomy (LSG).

We searched PubMed, MEDLINE, Cochrane Library, SCOPUS, and Web of Science for articles published in English from the first inception up to January 2022. Two researchers (SA and HM) screened the titles, abstracts, and references of the included studies for relevant articles; any discrepancy was solved by a consensus. The search was performed using combinations of bariatric surgery, sleeve gastrectomy, Roux-en-Y gastric bypass, diabetes, hypertension, dyslipidemia, gastroesophageal reflux, OSA, 5 years, and comorbidity resolution. We identified 2,457 studies, 1,468 of which stood after the removal of duplications; from them, 81 full texts were screened and only 16 studies were included in the final meta-analysis. A datasheet was used to extract the author's name, year, and country of publication, as well as the study type, and comorbidities remission (Tables 1, 2 and Figure 1).

**Table 1:** Randomized controlled trials comparing LRYGB and LSG regarding diabetes remission.

Author	Country	LRYGB	LSG	Results
Hofso et al. 2019 (14)	Norway	15/54	9/55	LRYGB better RR, RR] 1·57, 95% CI, 1·14–2·16, *P* = 0.0054
Kehagias et al. 2011 (15)	Greece	4/30	4/30	No significant difference
Keidar et al. 2013 (16)	Israel	9/19	14/18	No significant difference
Lee et al. 2011 (17)	Taiwan	13/16	3/16	LRYGB better, *P* = 0.05
Murphy et al. 2018 (18)	New Zealand	29/52	26/49	No difference in remission, *P* = 0.82
Peterli et al. 2018 (19)	Switzerland	19/104	16/101	No significant differences at 5 years, *P* = 0.77
Ruiz-Tovar et al. 2019 (20)	Spain	51/59	50/61	No significant difference
Salminen et al. 2018 (21)	Finland	18/40	15/41	No significant difference
Schauer et al. 2017 (22)	USA	15/49	11/47	Bypass better, *P* = 0.01
Tang et al. 2016 (23)	China	14/38	17/34	No significant difference, *P* = 0.19
Wallenius et al. 2020 (24)	Sweden	11/25	11/24	No significant differences at 1 and 2 years, *P* = 0.897
Wölnerhanssen et al. 2021 (25)	Switzerland & Finland	22/68	16/67	No significant differences at 1 and 2 years, 0.650
Yang et al. 2015 (26)	China	23/27	22/28	No significant difference, *P* = 0.525
Zhang et al. 2014 (27)	China	7/32	8/32	No significant difference
Shivakumar et al. 2018 (28)	India	74/100	72/100	No significant difference
Navarini et al. 2020 (29)	Brazil	19/28	4/24	LRYGB better

LRYGB, Roux-en-Y gastric bypass; LSG, laparoscopic sleeve gastrectomy.

**Table 2:** The quality of the included trials.

Author	Random bias	Allocation bias	Blinding of participants	Blinding of outcomes	Incomplete outcomes data	Selective reporting bias	Other bias
Hofso et al. 2019 (14)	Low	Low	Low	Low	Low	Low	Unclear
Kehagias et al. 2011 (15)	Low	Low	High	Unclear	High	Low	Unclear
Keidar et al. 2013 (16)	Low	High	High	Unclear	High	Low	Unclear
Lee et al. 2011 (17)	Low	Unclear	High	Unclear	High	Low	Unclear
Murphy et al. 2018 (18)	Low	Low	Unclear	Low	Low	Low	Unclear
Peterli et al. 2018 (19)	Low	Low	High	Unclear	Low	Low	Unclear
Ruiz-Tovar et al. 2019 (20)	Low	Low	High	Unclear	Low	Low	Unclear
Salminen et al. 2018 (21)	Low	Low	High	Low	Low	Low	Unclear
Schauer et al. 2017 (22)	Low	Low	High	Unclear	High	Low	Unclear
Tang et al. 2016 (23)	Low	Low	High	Unclear	Low	Low	Unclear
Wallenius et al. 2020 (24)	Low	Low	High	Low	Low	Unclear	Unclear
Wölnerhanssen et al. 2021 (25)	Low	Low	High	Low	Low	Unclear	Unclear
Yang et al. 2015 (26)	Low	Low	High	Unclear	Low	Unclear	Unclear
Zhang et al. 2014 (27)	Low	Low	High	Unclear	Low	Low	Unclear
Shivakumar et al. 2018 (28)	Low	Low	High	Low	Low	Low	Unclear
Navarini et al. 2020 (29)	Low	Unclear	High	High	Low	Low	Unclear

### Statistical analysis

The most recent version of the RevMan system (version 5, 4.) was used to compare LSG and LRYGB. The dichotomous data of 33 cohorts from 16 randomized controlled trials (14 studies comparing sleeve gastrectomy and Roux-en-Y gastric bypass on diabetes remission, 8 on hypertension, 5 on dyslipidemia, 4 on OSA, and 2 on gastroesophageal reflux) were entered manually. The odd ratio, 95% CI, and standard mean difference were measured. The Chi-square and *I*^2^ were used to quantify the heterogeneity, where the random effect was used if significant (dyslipidemia arm). Otherwise, the fixed effect was applied. A *P*-value of <0.05 was considered significant. A modified Cochrane risk of bias assessed the quality of the included studies (14). We strictly followed the standards of the Preferred Reporting Items for Meta-analysis (PRISMA) (15).

## Results

### Characteristics of the included trials

The author's name, country, year of publication, age, sex, BMI, duration of diabetes, waist circumference, HbA1c, and period of follow-up at baseline are depicted in Table 3. Diabetes remission only was reported in references (14, 16–18, 23, 24), diabetes and hypertension in (15, 19–22, 25–27), diabetes, hypertension, and dyslipidemia in (19–21, 25, 27). In addition, reference (19) reported all the ends and reference number (15) reported all ends except dyslipidemia. The current meta-analysis assessed the short (seven studies) and medium outcomes (nine studies). Regarding the diagnoses of diabetes mellitus, some of the studies followed the American guidelines (19, 21), while others were not (14, 17, 18, 20).

**Table 3:** Basic characters of the randomized controlled trials comparing LSG and LRYGB (LSG vs. LRYGB).

Author	Age	Women %	BMI	Diabetes duration	HbA1c	Waist circumference	Follow-up
Hofso et al. 2019 (14)	47·1 ± 10·2 vs. 48·2 ± 8·9	58% vs. 74%	42·1 ± 5·3 vs. 42·4 ± 5·4	6·3 ± 5·5 vs. 6·6 ± 6·5	7·9 vs. 7.6	128 ± 12 vs. 127 ± 12	1 year
Kehagias et al. 2011 (15)	36.0 ± 8.4 vs. 33.7 ± 9.9	66.7%	45.8 ± 3. 7 vs. 44.9 ± 3.4	NA	NA	NA	3 years
Keidar et al. 2013 (16)	47.7 vs. 51.45	43%	42.5 ± 5.2 vs. 42. ± 4.8	NA	NA	NA	1 year
Lee et al. 2011 (17)	45.8 ± 9.5 vs. 44.1 ± 8.4	71.8% women	31.5 ± 3.2 vs. 29.6 ± 3.2	NA	NA	NA	2 years
Murphy et al. 2018 (18)	45.5 ± 6.4 vs. 46.6 ± 6.7	45% vs. 59%	25–45 in 77% vs. 71%	<5 years in 41.4% vs. 46.4	61.9 ± 12.8 vs. 64.5 ± 18.1	NA	1 year
Peterli et al. 2018 (19)	43.0 ± 11.1 vs. 42.1 ± 11.2	72% vs. 71.8%	43.6 ± 5.2 vs. 44.2 ± 5.3	NA	NA	NA	5 years
Ruiz-Tovar et al. 2019 (20). HYT	43.9 ± 10.9 vs. 45 ± 11.3	75% both arms	45.3 ± 3.2 vs. 46.5 ± 3.4	NA	NA	NA	5 years
Salminen et al. 2018 (21). HYT	48.5 ± 9.6 vs. 48.4 ± 9.3	71.9% vs. 67.2%	45.5 ± 6.2 vs. 46.4 ± 5.9	NA	NA	NA	5 years
Schauer et al. 2017 (22)	49 ± 8	66% females	37 ± 3.5	NA	9.5 ± 1.7 vs. 9.3 ± 1.4	NA	5 years
Tang et al. 2016 (23)	36.6 ± 8.0 vs. 40.4 ± 12.3	64.7% vs. 47.4%	38.4 ± 8.6 vs. 37.8 ± 5.6	5.1 ± 4.1 vs. 6.5 ± 4.1	7.4 ± 1.8 vs. 7.4 ± 1.8	116.7 ± 19.2 vs. 113.3 ± 14.5	2 years
Wallenius et al. 2020 (24)	51.9 ± 1.9 vs. 51.2 ± 1.6	55.5% vs. 46.7%	36.9 ± 0.7 vs. 38.6 ± 0.8	6.5 ± 1.1 vs. 5.7 ± 0.6	55.7 ± 2.1 vs. 61.8 ± 3.9	NA	2 years
Wölnerhanssen et al. 2021 (25)	45.9 ± 10.7 vs. 45.3 ± 10.7	71.9% vs. 69.6%	45.6 ± 6.5 vs. 46.4 ± 6.6	NA	7.1 vs. 7	NA	5 years
Yang et al. 2015 (26)	40.4 ± 9.4 vs. 41.4 ± 9.3	71.9% vs. 59.4%	31.8 ± 3.0 vs. 32.3 ± 2.4	4.0 ± 1.7 vs. 4.2 ± 1.9	8.5 ± 1.2 vs. 8.9 ± 1.3	103.0 ± 7.7 vs. 104.5 ± 6.8	3 years
Zhang et al. 2014 (27)	29.3 ± 9.8 vs. 32.2 ± 9.2	62.5% vs. 56.2%	38.5 ± 4.2 vs. 39.3 ± 3.8	NA	NA	NA	5 years
Shivakumar et al. 2018 (28)	39.89 ± 11.75 vs. 42.89 ± 14.02	65% vs. 62%	44.57 ± 7.16 vs. 44.32 ± 7.88	NA	NA	NA	3 years
Navarini et al. 2020 (29)	39.3 ± 12.1	83% women	41.5 ± 5.1	NA	NA	NA	1 year

BMI, body mass index; LRYGB, Roux-en-Y gastric bypass; LSG, laparoscopic sleeve gastrectomy; NA, not applicable.

### Diabetes remission

**Figure 2:**
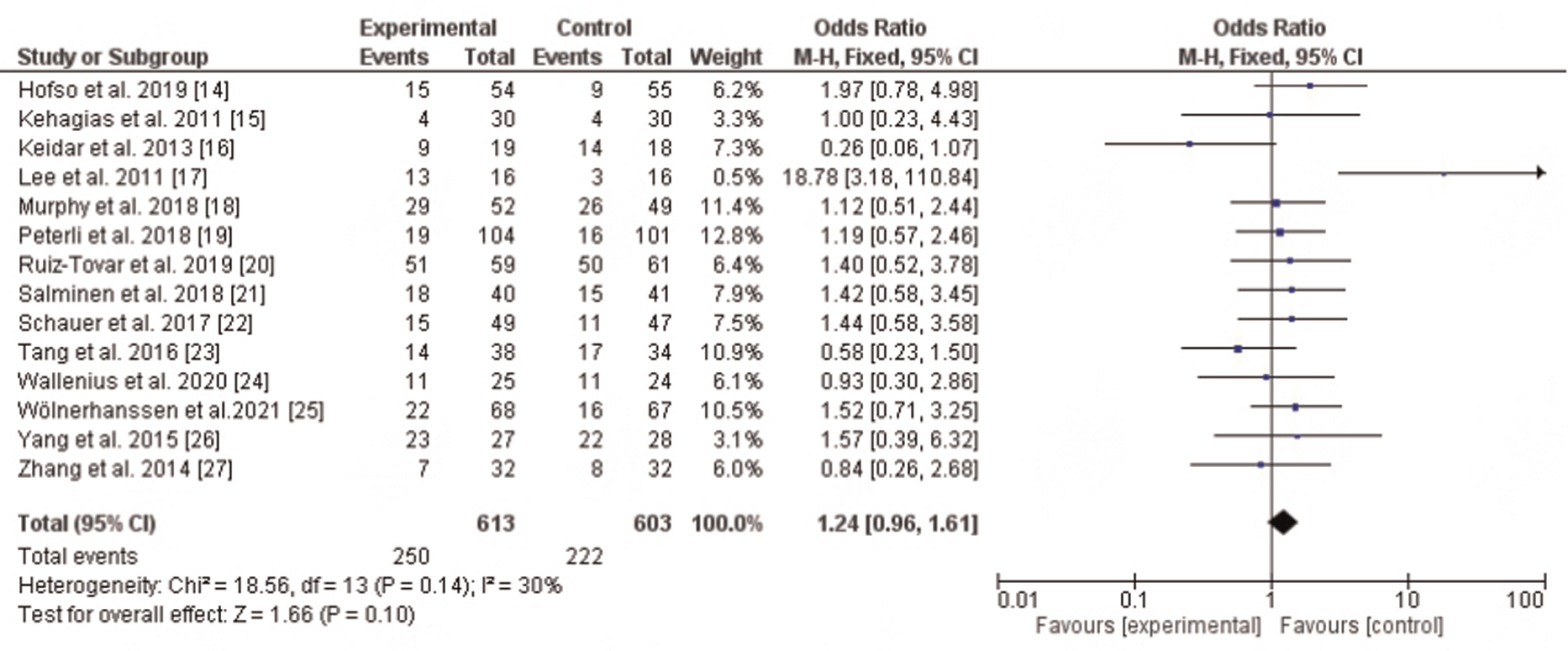
Diabetes remission following Roux-en-Y gastric bypass and sleeve gastrectomy.

In the present meta-analysis, 14 randomized controlled trials assessed diabetes remission following Roux-en-Y gastric bypass and laparoscopic sleeve gastrectomy (14–27). No significant statistical difference was found between LRYGB and LSG regarding diabetes remission, *P*-value = 0.10, odd ratio, 1.24, 95% CI, 0.96–1.61, *I*^2^ for heterogeneity = 30%, *P*-value for heterogeneity, 0.14 (Figure 2).

### Hypertension and dyslipidemia remission

**Figure 3:**
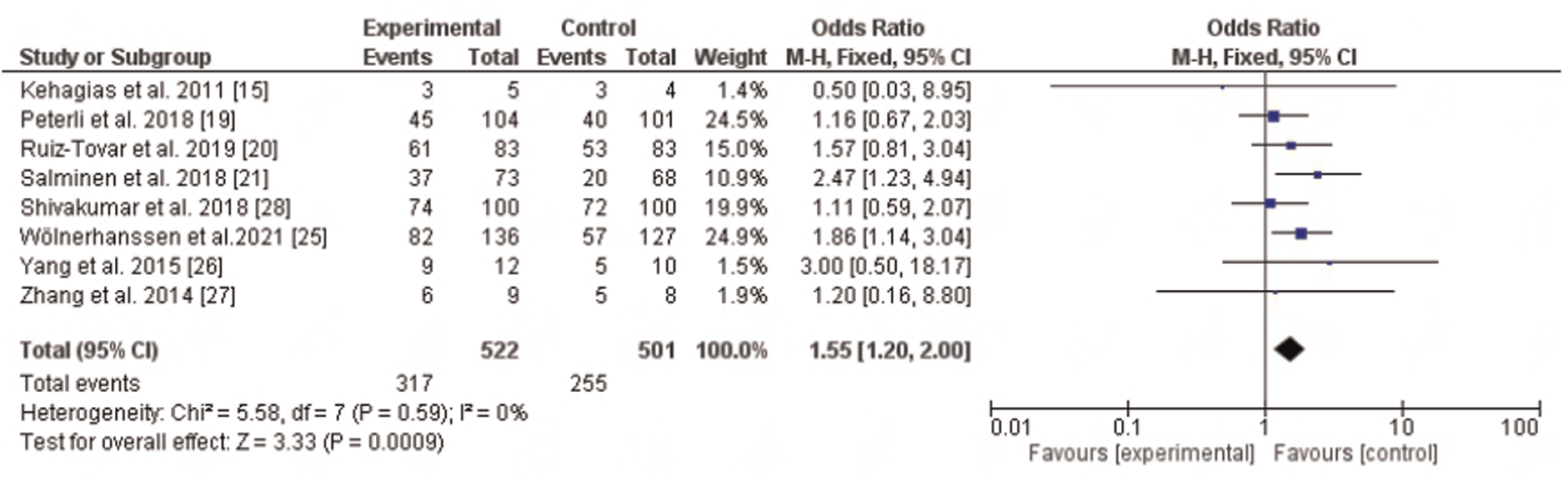
Hypertension remission following Roux-en-Y gastric bypass and sleeve gastrectomy.

**Figure 4:**
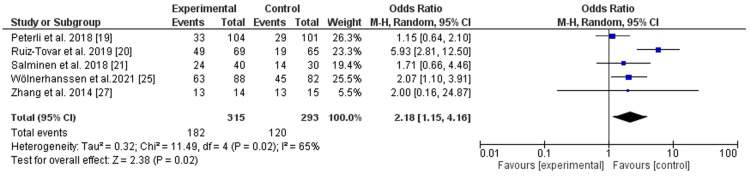
Dyslipidemia remission following Roux-en-Y gastric bypass and sleeve gastrectomy.

LRYGB was superior to LSG regarding hypertension remission (eight cohorts) (15, 19–21, 25–28) with significant statistical difference, *P*-value = 0.009, odd ratio, 1.55, 95% CI, 1.20–2.0, *I*^2^ for heterogeneity = 0.0%, *P*-value for heterogeneity, 0.59 (Figure 3). LRYGB was also superior for dyslipidemia remission (five cohorts) (19–21, 25, 27). However, substantial heterogeneity was observed, *I*^2^ = 65%, *P*-value for heterogeneity, 0.02, odd ratio, 2.18, 95% CI, 1.15–4.16, *P*-value for overall effect, 0.02 (Figure 4).

### Obstructive sleep apnea remission

**Figure 5:**
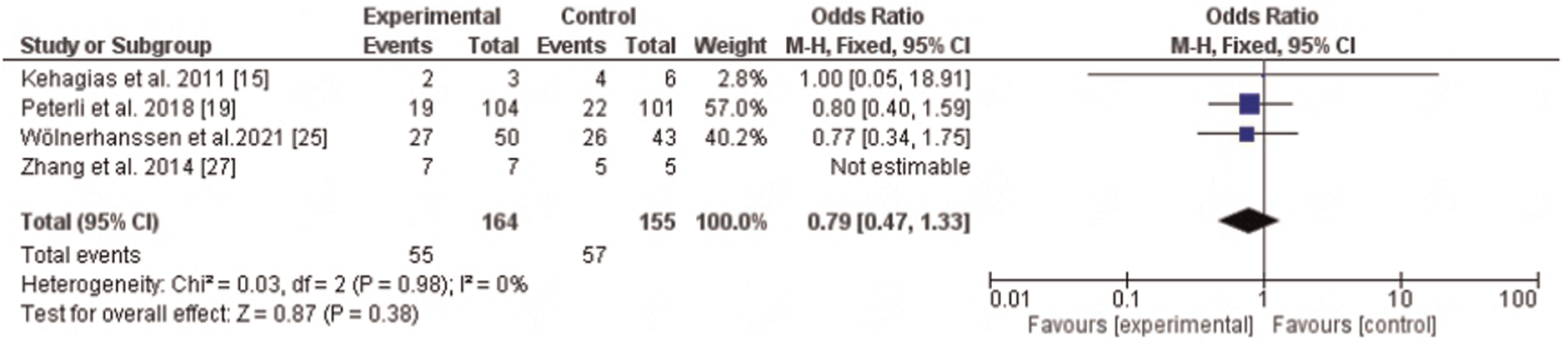
Obstructive sleep apnea remission following Roux-en-Y gastric bypass and sleeve gastrectomy.

Obstructive sleep apnea was assessed by four trials (15, 19, 25, 32), LSG was equal weight to LRYGB, *P*-value = 0.38, odd ratio, 0.79, 95% CI, 0.47–1.33, *I*^2^ for heterogeneity = 0.0%, *P*-value for heterogeneity, 0.98 (Figure 5).

### Gastroesophageal reflux remission and new-onset reflux following surgery

**Figure 6:**
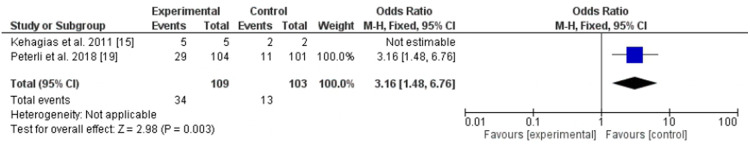
Gastroesophageal reflux remission following Roux-en-Y gastric bypass and sleeve gastrectomy.

**Figure 7:**
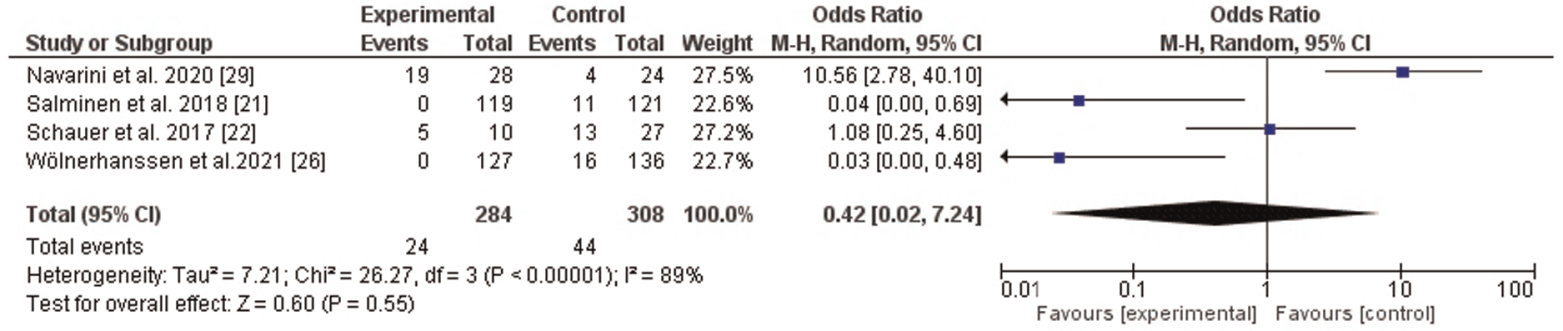
New-onset gastroesophageal reflux following Roux-en-Y gastric bypass and sleeve gastrectomy.

LRYGB was better for GERD remission (two trials) (15, 19), *P*-value = 0.003, odd ratio, 3.16, 95% CI, 1.48–6.76, heterogeneity not applicable. However, no significant statistical difference between LRYGB and LSG regarding the new-onset of GERD (four trials) (21, 22, 25, 28), *P*-value = 0.55, odd ratio, 0.42, 95% CI, 0.02–7.24, with substantial heterogeneity, *I*^2^ = 089%, *P*-value for heterogeneity, 0.0001 (Figures 6, 7).

## Discussion

### Diabetes remission after Roux-en-Y gastric bypass and sleeve gastrectomy

There is a recent shift in an indication of bariatric surgery from a certain BMI to a comorbidity-based approach where interdisciplinary care by surgeons, endocrinologists or internists, a psychologist, and a dietician is needed before surgery.

In the present meta-analysis, diabetes remission was similar between LRYGB and LSG in contradiction to Gu et al. (32). However, Gu and colleagues’ study was limited by pooling different methodologies and including only four trials. A recent meta-analysis (33) included 10 RCTs, which showed the superiority of LRYGB over LSG over the short term only. Another meta-analysis published by Sharples et al. (34) showed that LRYGB and LSG were similar regarding glycemic control at 5 years, in line with our findings. In addition, more reduction of serum cholesterol and lower gastrointestinal reflux were observed among patients following LRYGB in similarity to the present findings, however, the study included only four trials.

Higher levels of bile acids were observed in LRYGB patients with a similar rate of diabetes remission in LRYGB and LSG, indicating a role of BA in glycemic control (35).

Sha et al. (36) Lee et al. (37), Huang et al. (38), and Zhao et al. (39) meta-analyses concluded the similarity of LRYGB and LSG regarding diabetes remission. The previous studies were limited by pooling studies with different methodologies, a small number of the included studies, and a short period of follow-up.

### The effects on hypertension and dyslipidemias

The effects of LRYGB and LSG on hypertension and dyslipidemia were discussed controversially. In addition, all the meta-analyses were limited by pooling different methodologies and a small sample of well-randomized studies. A meta-analysis of 14 studies showed no difference regarding the resolution of hypertension and improvement of cholesterol and triglycerides (40); further meta-analysis supported these findings (41, 42) in contradiction to Gu et al. (32) Li et al. (43) and Zhao et al., who showed the superiority of LRYGB. In the present study, LRYGB was superior regarding hypertension and dyslipidemia remission. The current findings were consistent with Climent et al. who showed a more sustained effect of LRYGB on blood pressure (44). A meta-analysis of randomized controlled trials (45) showed a higher resolution of dyslipidemia with a high certainty at 1 year and moderate evidence at 3 years with no differences regarding hypertension.

### Gastroesophageal reflux

The evidence regarding gastroesophageal reflux is largely based on observational studies and systematic reviews. A retrospective analysis with a large sample size showed that LSG is associated with more gastroesophageal reflux; indeed 16% need conversion to LRYGB at 10 years (46). More retrospective studies showed similar observations (47). Recent systematic reviews showed a higher rate of GERD among patients with LSG (48, 49). A retrospective analysis showed that conversion to LRYGB is an effective approach for those with significant GERD (48). A randomized controlled trial concluded the superiority of LRYGB for the treatment of GERD (50). Thus, LRYGB is better for those with GERD.

### Obstructive sleep apnea

Bariatric surgery poses beneficial effects on pulmonary function; a previous study showed that 48% and 80% of patients with bronchial asthma and OSA were symptom-free at 5 years, and another study showed remission/improvement in 90% and 90.74% (51, 52). Regarding OSA, no differences in remission were noted regarding the type of operation (53–55) in line with the present study in which LRG and LRYGB were similar.

### Surgical complications

In LRYGB, a gastric pouch was performed by dividing the stomach with a linear stapler and then gastrojejunal anastomosis was created. LSG was conducted by dividing the stomach with the linear stapler through the large gastric curvature; resection was completed and running imbricated absorbable suture (29).

Sleeve gastrectomy is associated with fewer early and late postoperative complications and reoperation when compared to LRYGB, Sha et al. (36) reported a higher dumping syndrome among patients after LRYGB. The operation time is an advantage for LSG (27–29, 32–41, 56). Furthermore, LSG showed lower rates of iron and vitamin D deficiency, and fewer calcium and phosphorus abnormalities (57, 58). Postoperative bleeding was lower in LSG (59). On the other hand, LRYGB was associated with lower rates of gastroesophageal reflux (60).

### Effects on gut hormones

The effects of LSG and LRYGB on gut hormones are complex and inconsistent. Some studies showed no difference in body weight between LSG and LRYGB (61, 62). Although the great effects of LRYGB on body weight compared to LSG might be mediated in part through gut hormones (32, 45, 63).

LSG showed lower Ghrelin and higher resistin than LRYGB, with no differences regarding glucagon-like peptide-1, gastric inhibitory peptide, and leptin (17). Gu et al. (64) found a significant reduction in fasting peptide YY among patients following LRYGB, while ghrelin was reduced after LSG. A great area under the curve of fasting peptide YY and increased glucagon-like peptide-1 was observed among patients with LRYGB (65). Other studies found no differences between the two procedures regarding glucagon-like peptide-1, gastric inhibitory peptide (66).

This manuscript gave insight into patients' categorization before bariatric surgery and choosing the suitable type of surgery according to comorbidities. However, the small number of studies included, the heterogeneity observed in the dyslipidemia arm, and the discrepancy in the diagnosis of ends and remission are limitations.

### Nonalcoholic fatty liver disease (NAFLD)

Bariatric surgery is beneficial for nonalcoholic fatty liver disease (NAFLD), recently renamed metabolic-related fatty liver disease. The mechanism involves bile acid pathways, gut hormones, and intestinal microbiota. The data on the effects of LRYGB and LSG on liver enzymes are scarce; however, LSG seems better at normalizing liver enzymes in 1 year (67, 68).

## Conclusion

LRYGB was superior to LSG for gastroesophageal reflux, hypertension, and dyslipidemia remission. While the two procedures were equal regarding diabetes and obstructive sleep, LSG may be better for metabolic-related fatty liver disease and is associated with fewer surgical complications. Further reviews comparing LSG and one anastomosis gastric bypass are recommended.

## Data Availability

The raw data supporting the conclusions of this article will be made available by the authors, without undue reservation.
